# A Mechanical–Electrochemical Dual-Model E-Skin for the Monitoring of Cardiovascular Healthcare

**DOI:** 10.3390/bios15010005

**Published:** 2024-12-26

**Authors:** Jianxiao Fang, Yunting Jia, Zelong Liao, Bairui Qi, Tao Huang

**Affiliations:** 1 Department of Materials Science and Engineering, Southern University of Science and Technology, Shenzhen 518055, China; jianxiao.fang@student.manchester.ac.uk (J.F.); 12210854@mail.sustech.edu.cn (Y.J.); 12232028@mail.sustech.edu.cn (Z.L.); 2 Department of Materials Science and Engineering, Central South University, Changsha 410004, China; bairuiqi1026@outlook.com; 3 Department of Materials, The University of Manchester, Manchester M13 9WL, UK

**Keywords:** mechano-sensing, electrochemical sensing, dual-model electronic skin, iontronic structure, ion-selective electrode

## Abstract

The early monitoring of cardiovascular biomarkers is essential for the prevention and management of some cardiovascular diseases. Here, we present a novel, compact, and highly integrated skin electrode as a mechanical–electrochemical dual-model E-skin, designed for the real-time monitoring of heart rate and sweat ion concentration, two critical parameters for assessing cardiovascular health. As a pressure sensor, this E-skin is suitable for accurate heart rate monitoring, as it exhibits high sensitivity (25.2 pF·kPa^−1^), a low detection limit of 6 Pa, and a rapid response time of ~20 ms, which is attributed to the iontronic sensing interface between the skin and the electrode. Additionally, the electrode functions as a potassium ion-selective electrode based on chemical doping, achieving an enhanced response of 11 mV·mM^−1^. A test based on the real-time monitoring of a subject riding an indoor bike demonstrated the device’s capability to monitor heart rate and sweat potassium ion levels reliably and accurately. This advancement in wearable technology offers significant potential for enhancing patient care based on the early detection and proactive management of cardiovascular conditions.

## 1. Introduction

Cardiovascular diseases (CVDs) remain among the most prevalent and fatal health conditions globally, affecting millions annually. The early monitoring of cardiovascular health markers, such as pulse rate and sweat ion concentrations, is vital for CVD prevention and patient outcomes [1,2,3]. Recent advancements in wearable electronics, particularly skin-integrated devices, have enabled continuous on-body bio-signal monitoring during daily activities [4,5,6,7,8,9,10,11,12]. However, despite ongoing improvements in electrical systems and signal processing for cardiovascular monitoring, significant barriers to widespread clinical adoption remain. A major challenge lies in balancing the simplicity of monitoring systems with the complexity of multi-modal signal detection, which can lead to user discomfort and degraded signal quality, ultimately reducing diagnostic accuracy.

Current methods for monitoring physiological parameters—including heart rate variability [13], pulse wave velocity [14], and sweat potassium ion concentrations [15]—often necessitate multiple wearable devices, complicating patient compliance. Recently, innovative concepts featuring highly integrated multi-mode sensors have gained more attention, particularly those utilizing miniaturized functionalized electrochemical electrodes for simultaneous biomarker monitoring [16,17]. Nonetheless, there are few studies focusing on cross-disciplinary signals simultaneously, for example, mechanical biosignals and electrochemical markers. Challenges in material compatibility, device configurations, and interfacial management still hinder progress in this field.

**Figure 1:**
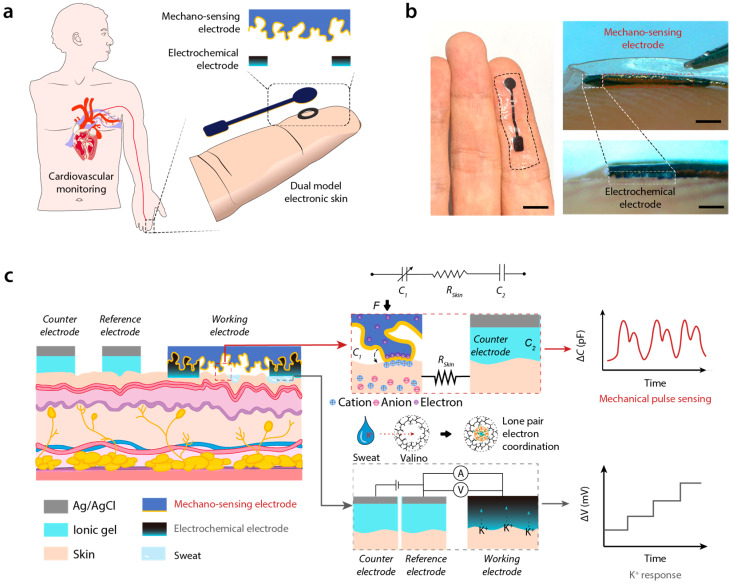
Brief introduction and working principle of DMSE-skin. (**a**) Schematic description of DMSE-skin’s structure and its possible usage in CVD monitoring; (**b**) photos showing the DMSE-skin on the skin and the thickness of different layers; (**c**) working principles of mechano-sensing for pulse and electrochemical sensing for K^+^ recording.

In this study, we developed dual-model sensing electronic skin (DMSE-skin) designed for the integrated detection of mechanical pulse sensing and metabolic sweat ion concentrations. DMSE-skin is applied as a patch to the human skin (Figure 1a), utilizing microstructured electrodes to form pressure-dependent iontronic interfaces for high-sensitivity mechanical sensing. A functionalized spacer, acting as an electrochemical electrode, is strategically positioned between the main electrode and the skin to ensure stable sensing properties and enable precise ion detection. The flexible substrate houses the sensors and provides both comfort and reliable performance. Our design enables the precise and non-invasive measurement of critical cardiovascular indicators, such as pulse rate, blood pressure, and sweat potassium concentration. This advancement in combined mechanical and electrochemical sensing marks a significant step forward in the evolution of biosensors and wearable electronic technologies.

## 2. Materials and Methods

Materials used: Sylgard 184 Silicone Elastomer Base from Dow, Midland, MI, USA; Sylgard 184 Silicone Elastomer Curing Agent from Dow, Midland, MI, USA; PEDOT:PSS water solution from Xi’an Yuri Solar Co., Ltd., Xian, China; Waterborne Polyurethane (WPU, ~20 wt.%) from Macklin, Shanghai, China; Silicone Rubber (types 708) from Liyang Hongda Rubber Industry Co., Ltd., Liyang, China; Dimethyl Sulfoxide (DMSO, A.R.) from Macklin, Shanghai, China; Potassium Chloride (KCl, 99.5% purity, A.R.) from Aladdin, Shanghai, China; Valinomycin ≥ 98% (TLC), ≥95% (HPLC), Potassium Tetraphenylborate (KTCPB) ≥ 98.0% (HPLC) from Macklin, Shanghai, China; Phosphate-buffered saline (PBS) from Servicebio (G4202), Wuhan, China.

Instruments used: Jinghong Vacuum Drying Oven, 2XZ-2 Rotary Vane Vacuum Pump from Zhejiang Taizhou Qiu Jing Vacuum Pump Co., Ltd., Taizhou, China; Thinky S1903553 Rotation/Revolution Mixer from Thinky, Tokyo, Japan; Lichen Hot Plate from Zhejiang Lichen Instrument Technology Co., Ltd., Shaoxing, China; Kurt J. Lesker Magnetron Sputtering Platform from Kurt J. Lesker Company, Pittsburgh, Pennsylvania, USA, HITACHI Ion Sputtering Platform from Hitachi High-Tech Corporation, Tokyo, Japan; HUPL-300D UV Laser Cutting Machine was applied from Shenzhen Chaoyue Laser Intelligent Equipment Co., Ltd., Shenzhen, China; Pressure was controlled by XLD-20E force gauge from Guangzhou Jingkong Testing Instruments Co., Ltd., Guangzhou, China; Morphology of microstructure was observed by FE-SEM from Tescan HITACHI, Tokyo, Japan; Capacitance was measured by inductance-capacitance-resistance (LCR) meter (E4980AL, Keysight Technologies), Santa Rosa, CA, USA; Impedance and potential were measured by CS350H electrochemical workstation from Wuhan CorrTest Instruments Corp., Ltd., Wuhan, China.

Fabrication of sensors: A glass sheet was chosen and attached with double-sided tape to one side. The sandpaper of varying meshes, like 60, 240, 280, and 5000, was adhered on the taped glass surface as the mold. The PDMS precursor is prepared by mixing Sylgard 184 silicone elastomer base with its curing agent at a standard ratio of 10:1 by weight. This mixture needs to be thoroughly degassed to remove air bubbles. After degassing, the PDMS mixture was carefully poured into the prepared mold. The mold with PDMS precursor was then placed on a hot plate set at 70 °C and left to cure for 2 to 3 h. Afterwards, the PDMS film with sandpaper structure is demolded carefully.

Next, a solution was prepared by mixing 8 g of PEDOT:PSS (1 wt.%), 2 g of WPU (20 wt.%), and a drop of surfactant (FS-30). The prepared solution is then centrifuged to ensure it is homogeneous. The resulting homogeneous solution was then evenly poured onto the PDMS mold. The solution on the mold was heated on a hot plate set at 60 °C for 2 h, followed by an overnight baking process in an oven set at 80 °C. The dried PEDOT:PSS-WPU microstructured film was then peeled off and sputtered with 10 nm Ti and 100 nm Au. The sputtered PEDOT:PSS-WPU electrode was then laser cut into desired shapes.

To prepare the spacer or electrochemical electrode, two separate solutions are mixed in different vials. A mixture contained 6 g of polyurethane /ethanol solution (30 wt.%), 2 g of PEDOT:PSS (1 wt.%), 0.6 g of DMSO, and 10% to total mass of valinomycin and KTCPB (5% to total mass) (Supplementary Figure S1) [18]. The mixture was stirred thoroughly until a uniform mixture was achieved. Afterward, it was poured into a flat Teflon mold and dried at 80 °C. The resulting film typically has a thickness of around 40 µm. After the film is cured, it is carefully removed from the mold, and a laser-cutting machine is used to cut the film into circular rings. Before the spacer is affixed to the substrate, it undergoes an immersion process in a 0.01 M KCl solution for 2 h.

The circular ring spacer was precisely aligned with the electrode’s circular area and affixed using ethanol. The central section of the electrode was then covered with insulating adhesive to protect the conductive pathways from external interference.

## 3. Results

### 3.1. Brief Introduction and Working Principle of DMSE-Skin

The dual-mode sensing electronic skin (DMSE-skin) sensor integrates pressure-induced mechanosensing for pulse monitoring with electrochemical sensing for sweat ion concentration detection. The sensor consists of two distinct layers (Figure 1b): an upper mechanosensing electrode approximately 100 μm thick and 5 mm in diameter and a middle electrochemical sensing electrode that is about 40 μm thick and has an annular shape featuring an inner diameter of 3.5 mm and an outer diameter of 4.5 mm. Designed for direct attachment to the fingertip, the sensor is securely attached to the skin with medical adhesive PU tape (3M Tegaderm). When applied to other knuckles, it reliably monitors pulse wave signals by using a capacitance meter (Supplementary Figure S2). The overall thickness of the patch is approximately 150 μm, ensuring comfortable wear.

The upper mechano-sensing electrode is made of a stretchable elastomer composed of commercial water-borne polyurethane and poly (3,4-ethylenedioxythiophene): polystyrene sulfonate (PEDOT:PSS). Its microstructure, templated by 240-mesh sandpaper, demonstrated superior performance compared with other mesh sizes (Supplementary Figure S3). A 100 nm layer of gold was sputtered onto the mechano-sensing electrode to enhance conductivity (Supplementary Figure S4). The middle layer comprises PEDOT:PSS, WPU, and valinomycin (a potassium ion-selective reagent). All layers are integrated via strong intermolecular interactions. The electrode fabrication procedure, along with side and overview images of the electrode’s surface microstructure, is detailed in Supplementary Figure S5.

The operational principle of the DMSE-skin sensor is based on two primary mechanisms. For pressure sensing, when the microstructured mechano-sensing electrode comes into contact with the human skin, the electrons in the electrode and the ions in the skin form a non-Faradaic junction behaving like a supercapacitor [4]. When pressure is applied, the electrode-skin interface exhibits capacitance at the nanofarad level, which varies according to the pressure-dependent skin-electrode contact area. The microstructured surface of the electrode provides a finely tunable iontronic interface, enhancing sensitivity significantly. A counter electrode composed of Ag/AgCl ionic gel is conformably laminated onto the skin and connected in series with the sensing electrode (Figure 1c), enabling the detection of mechanical signals, such as cardiac pulsations, based on capacitance changes.

Furthermore, the electrochemical electrode between the mechano-sensing electrode and the skin is selective for potassium ions. Valinomycin, a cyclic peptide with a hollow-ring structure, features an internal cavity that precisely accommodates potassium ions. When a potassium ion enters this cavity, the oxygen atoms in valinomycin’s inner ring form electrostatic bonds with it by donating six lone pair electrons [19]. This interaction results in alterations in the electrochemical potential relative to the counter electrodes, which are detected with an electrochemical workstation, providing direct measurements of potassium ion concentration (Figure 1c). Additionally, this layer also functions as a spacer for the pressure-sensing mechanism, enhancing stability and performance. The entire electrode was attached to the skin of a volunteer for over 3 days, and the skin showed no significant signs of inflammation or discomfort (Supplementary Figure S6).

### 3.2. Mechano-Sensing Performance

**Figure 2:**
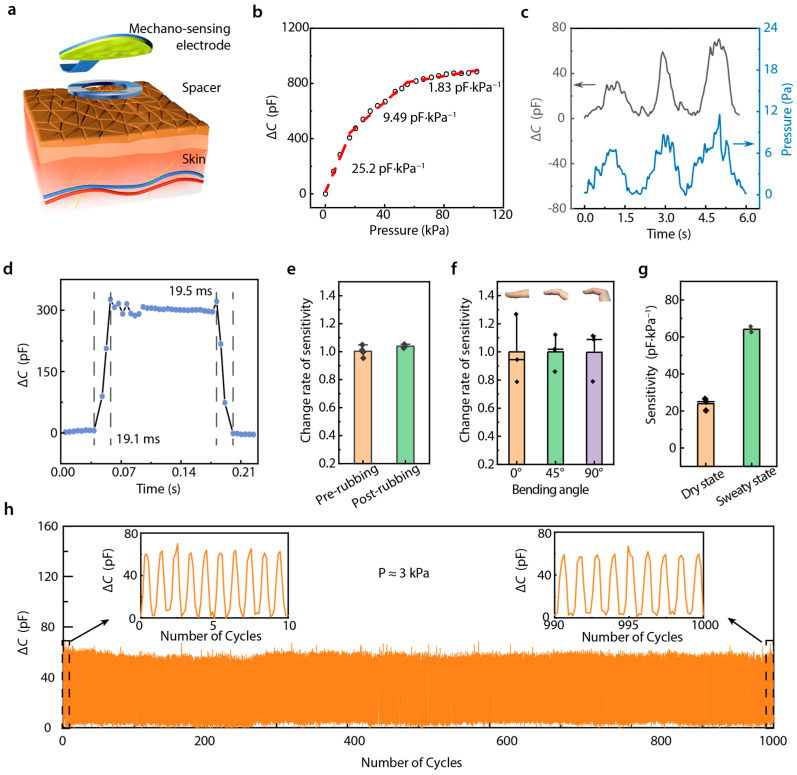
Mechano-sensing performance. (**a**) Diagram showing the sensing iontronic structure; (**b**) Δ*C*-*P* response curve of this sensor; (**c**) limit of detection (~6 Pa); (**d**) response time and recovery time; (**e**) change rate of sensitivity before and after rubbing with sandpaper; (**f**) change rate of sensitivity under 0°, 45°, and 90° bending angle on finger skin; (**g**) variation in sensitivity of sensors on the dry and sweaty finger skin; (**h**) cyclic loading–unloading of Δ*C*-*P* response under 3 kPa.

Our mechano-sensing process relies on the interaction between a microstructured electrode and ionic skin (Figure 2a). To illustrate the performance of this sensing structure, we characterized the relationship between capacitance change and applied pressure response in our DMSE-skin. The slope of the capacitance change-pressure curve is defined as sensitivity S = δ (Δ*C*)/δ*P*, where Δ*C* represents the change in capacitance, and *P* denotes the applied pressure. As shown in Figure 2b, our pressure sensor demonstrated a linear sensitivity of 25.2 pF·kPa^–1^ within a pressure range of approximately 0 to 20 kPa. At higher applied pressures, its sensitivity decreased to 9.49 pF·kPa^–1^ from 20 to 60 kPa and subsequently stabilized at 1.83 pF·kPa^–1^ from 60 to 100 kPa. Compared with previous works, our DMSE-skin sensors demonstrate competitive sensitivity (Supplementary Figure S7). The electrochemical electrode serves not only as a potassium ion detection electrode but also as a spacer that minimizes the initial contact between the microstructure and the skin. This dual functionality enhances sensitivity and improves signal output.

Pulse pressure typically falls within 1 kPa [20], requiring our mechanical sensor to exhibit an exceptionally low limit of detection (LOD). Our tests revealed that the LOD of our sensing structure was as low as 6.2 Pa (Figure 2c), which is significantly lower than the typical pressure of human skin (~1 kPa), indicating enhanced signal quality for cardiovascular monitoring. Typically, heart rates range from 50 to 200 beats per minute, necessitating sensors with rapid response and recovery times to accurately record pulse wave signals. As shown in Figure 2d, our sensing structure demonstrated response and recovery times under 20 ms, thereby enabling the precise and clear detection of individual pulse beats.

The stability of a sensor is also a vital factor in long-term usability. Variations in temperature cause the signal to change; however, on account of the essentially constant skin temperature, the pulse can still be tested with our DMSE-skin (Supplementary Figure S8). Additionally, sensors placed on the fingertips are often attached to rough surfaces, which can potentially influence their sensitivity. Therefore, we used 1000-mesh sandpaper to rub the sensor multiple times and measured the Δ*C-P* response. As shown in Figure 2e, after 100 rubbing cycles, the sensor maintained almost the same sensitivity level. We further evaluated the Δ*C-P* response in various finger-bending states, and the results showed that our sensors exhibited almost the same sensitivity in 0–90° bending states (Figure 2f), demonstrating robust performance under deformation. Additionally, sweating was found to increase the sensor’s sensitivity (Figure 2g). This can be ascribed to the increase in ion concentration on the skin during perspiration. Importantly, this increase in sensitivity was shown to exert no influence on pulse recording accuracy. Furthermore, the sensing structure could stably record during thousands of loading–unloading cycles (Figure 2h), which supports its potential long-term and multiple-time use in diverse real-world scenarios.

### 3.3. Potassium Ion Sensing Performance

**Figure 3:**
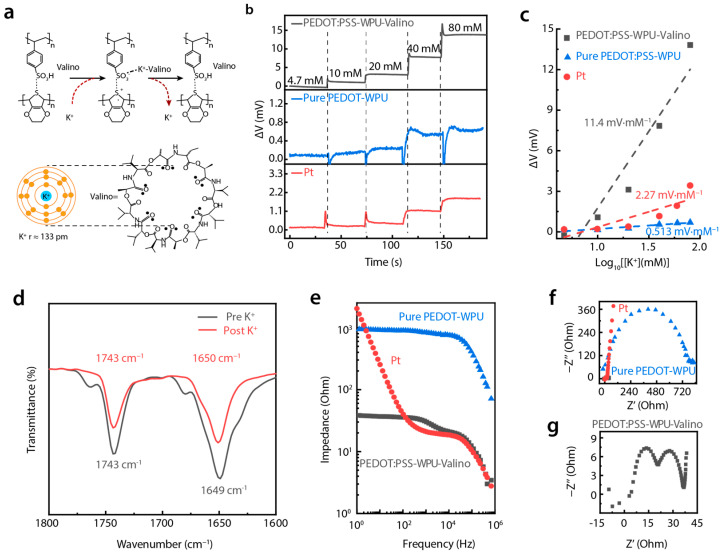
Potassium ion sensing performance. (**a**) Diagram illustrating the K^+^ sensing mechanism; (**b**) potential variation versus time curves of PEDOT:PSS-WPU-Valino, PEDOT:PSS-WPU, and Pt electrode in PBS solution with different addition of K^+^; (**c**) fitted potential variation versus log K^+^ concentration; (**d**) FT-IR results of valinomycin before and after absorption of K^+^; (**e**) Bode plot and (**f**) Nyquist plot of PEDOT:PSS-WPU-Valino, PEDOT:PSS-WPU, and Pt electrodes; (**g**) enlarged nyquist plot of PEDOT:PSS-WPU-Valino electrode.

Potassium ion (K⁺) concentration in sweat is a vital marker of cardiovascular health. In order to monitor by using our sensing structure, the typical insulative spacer found in the iontronic sensing mechanism was replaced with PEDOT:PSS-based conductive materials, which also function as an electrochemical electrode. In previous studies, it was reported that K⁺ doping enhanced the charge transfer capability of PEDOT:PSS [21,22]. However, PEDOT:PSS has limitations in rapidly capturing K⁺ ions alone, presenting challenges for real-time K⁺ detection. To address this, we designed a valinomycin-doped PEDOT:PSS-WPU electrode for K^+^-sensitive electrochemical detection. Valinomycin, a cyclic antibiotic molecule, exhibits a unique selectivity for K⁺ [23]. As depicted in Figure 3a, when K^+^ is captured by valinomycin, the K^+^-valinomycin compound interacts with the sulfone groups and releases more protons, which further enhance doping within the PEDOT-conjugated structure, leading to increased conductivity and alterations in electrochemical potential.

We evaluated the K^+^ concentration–polarization potential response (K^+^-V) by using the valinomycin-doped PEDOT:PSS-WPU electrode (PEDOT:PSS-WPU-Valino), a PEDOT:PSS-WPU electrode, and normal platinum (Pt) electrode in Phosphate-Buffer-Solution (PBS) with Pt as the counter electrode and Hg/HgCl_2_ as the reference electrode. As shown in Figure 3b, the PEDOT:PSS-WPU-Valino electrode exhibited the highest K^+^-V response amplitude. For example, the K^+^-V response of PEDOT:PSS-WPU-Valino at a K^+^ concentration of 20 mM was approximately 10 and 13 times greater than those of the PEDOT:PSS-WPU electrode and Pt electrodes, respectively, underscoring the better performance of our material. The sensitivity of the electrodes, calculated as the slope of potential change versus the logarithm of the K^+^ concentration, further highlights this advantage. As shown in Figure 3c, the PEDOT:PSS-WPU-Valino electrode achieved a sensitivity of 11.4 mV·mM^−1^, which is significantly higher than that of PEDOT:PSS-WPU (0.513 mV·mM^−1^) and Pt (2.27 mV·mM^−1^) electrodes. Moreover, the PEDOT:PSS-WPU-Valino electrode demonstrated excellent selectivity for K^+^ compared with Na^+^, lactic acid, glucose, and other metabolites in sweat (Supplementary Figure S9).

To further elucidate the fundamental process of K⁺ absorption in valinomycin, Fourier-transform infrared (FT-IR) spectroscopy was applied to analyze the electrode before and after its immersion in 1 M KCl-PBS. As shown in Figure 3d, the characteristic wavenumbers at 1743 cm^−1^ and 1649 cm^−1^ correspond to the tensile vibrations of ester-carbonyl and amido-carbonyl in valinomycin before K^+^ absorption (black curve). After coordination between the amide-carbonyl oxygen and K^+^, the hydrogen bond involved in the original amido-carbonyl group is either weakened or disrupted, resulting in a subtle blue shift in the amido-carbonyl signals and a noticeable reduction in absorption intensity. The interactions between K+ and valinomycin proved with FT-IR clarified the variation in the chemical structure of the film. However, the significant potential change remained unexplained.

To gain deeper insights into these potential changes, electrochemical impedance spectroscopy was employed to characterize the impedance of the electrode. Figure 3e shows the Bode plots of the PEDOT:PSS-WPU, PEDOT:PSS-WPU-Valino, and Pt electrodes in a KCl-containing PBS. While K^+^ could dope PEDOT:PSS itself, the impedance of the PEDOT:PSS-WPU electrode without valinomycin exceeded 8 × 10^2^ Ω in the frequency range of 1–10^4^ Hz. After the PEDOT:PSS-WPU was doped with valinomycin, due to the easy integration of K^+^, its impedance was less than 40 Ω in the frequency range of 1–10^4^ Hz, which is significantly lower than that of the Pt electrode. The valinomycin in the film functions as a selective tunnel and carrier of K^+^ with lower transfer energy. This specialized channel can significantly increase the mobility of K^+^, thereby reducing the overall impedance of the membrane.

The Nyquist plots in Figure 3f further corroborate these findings. The data indicate that the radius for PEDOT:PSS-WPU-Valino (black dots) is considerably lower than those of the PEDOT-WPU (blue dots) and Pt (red dots) electrodes, reflecting the lower charge-transfer resistance of the PEDOT:PSS-WPU-Valino electrode. Moreover, the Nyquist plot for the latter reveals two distinct semicircles (Figure 3g), reflecting multiple electrochemical processes during the impedance test. The first semicircle corresponds to charge transfer resistance. With the K^+^ selective response, the velocity of charge transfer increases, resulting in lower impedance and, thus, a lower curve for the K^+^ selective film. The second semicircle relates to the ion migration and adsorption/desorption process [24]. It is believed that the migration and adsorption of K^+^ in the electrode are dominated due to selective capture by valinomycin, which would cause the second semicircle. This indicates that the addition of valinomycin increases the electrochemical responses to K^+^, such as improving the adsorption and migration of K^+^ towards the film, which causes a more pronounced potential variation with changes in K^+^ concentration.

### 3.4. Robustness Against Interference of DMSE-Skin

**Figure 4:**
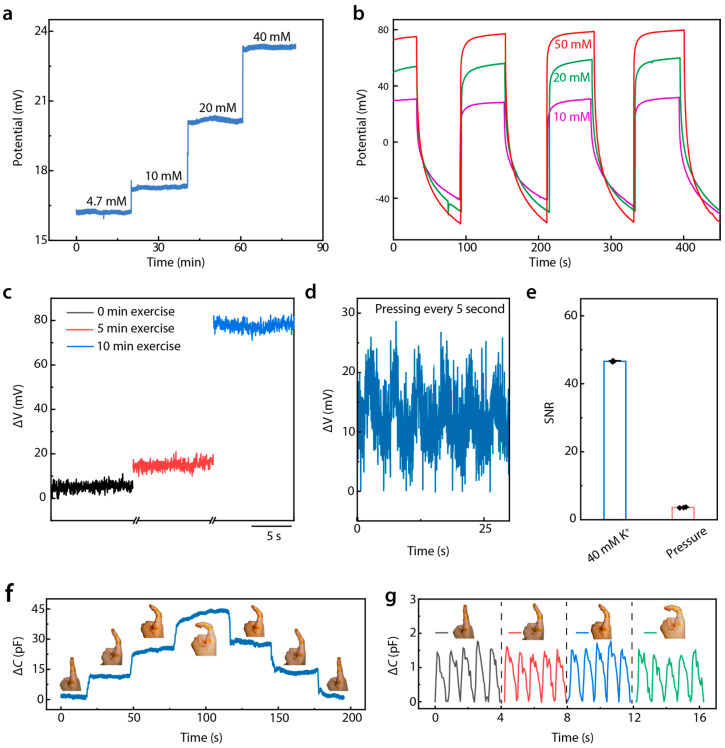
Immunity from the interference of DMSE-skin. (**a**) Long-time K^+^ response stability of the sensor; (**b**) repeatability under different K^+^ concentrations during perspiration; (**c**) potential variation in different periods of exercise; (**d**) potential responses of the sensor with pressing every 5 s; (**e**) signal-to-noise ratio of signals generated by sweat and pressing; (**f**) signal under different bending angle; (**g**) enlarged view of pulse wave signal under different bending angle.

For wearable and biocompatible devices, ensuring consistency and avoiding interference are critical requirements. Figure 4a demonstrates that the device could maintain a stable voltage response over extended periods (over 20 min) at different K^+^ concentrations (4.7 mM, 10 mM, 20 mM, and 40 mM). Body motion was found to have a negligible impact on potential changes (Supplementary Figure S10). This long-term consistency confirms the sensor’s reliability for continuous health monitoring without signal degradation. It is noticeable that the potential value increased gradually with an increase in temperature from 0 to 50 °C (Supplementary Figure S11). However, E-skin is applied to the skin, where the temperature stays around 36 °C, which ensures signal stability. As shown in Figure 4b, we further validated the device’s stability with repeated-usage tests. Exposing the sensor to multiple cycles at different K^+^ concentrations resulted in minimal variation, demonstrating excellent reproducibility and confirming that the sensor can withstand repeated use without significant accuracy loss.

To evaluate the device’s sensitivity to physiological changes, an exercise-based study was conducted (Figure 4c). The potential was measured after 0, 5, and 10 min of exercise, where the skin secreted variable degrees of sweat. Figure 4d presents that the sensor effectively tracked changes in K^+^ concentration due to sweat generated during physical exertion, underscoring its utility for the real-time monitoring of electrolyte balance in athletes or patients with cardiovascular concerns. To assess the interference between mechanical pressure and electrochemical potential, the sensor’s potential response was tested at a pressure of 50 kPa applied every 5 s. The voltage response remained consistent across repeated cycles of deformation and relaxation, indicating that mechanical deformation did not affect K^+^ concentration measurements significantly. Comparing the signal-to-noise ratio (SNR) of the electrochemical potential response generated during sweating with those caused by pressure (Figure 4e) revealed a significantly higher SNR for electrochemical signals, which further confirms that mechanical deformation does not significantly affect the measurement of K^+^ concentration. This demonstrates that external forces, such as pressing or bending, do not interfere with the sensor’s ability to accurately measure K^+^ concentration during perspiration.

The device’s ability to detect pulse wave signals under motion conditions was tested, such as finger bending, as shown in Figure 4f,g. Despite changes in the curvature of the finger, which induced mechanical stress on the sensor, the pulse signals remained clearly detectable and stable. The zoomed-in view in Figure 4g confirms that even under significant bending, the pulse signals maintained clarity. Since pulse signals are limited to the 5 pF amplitude and motion-caused signals are mostly in the order of tens of hundreds of picofarads, pulse signals can be calculated clearly with an electrical circuit or algorithm design. (Supplementary Figure S12) These findings demonstrate that our sensor provides stable, reliable, and repeatable measurements of sweat K^+^ concentrations and pulse signals under diverse conditions. These attributes position the device as a promising candidate for future applications in continuous, non-invasive health monitoring, especially for cardiovascular health management.

### 3.5. Application of DMSE-Skin

**Figure 5:**
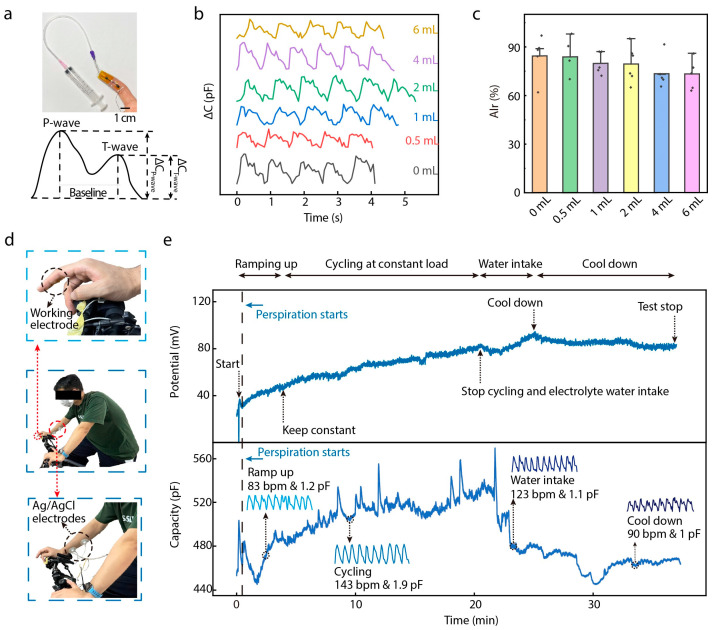
Application of DMSE-skin. (**a**) Photos of the sensor on fingertip under different preload. The inserting showing the ideal curve for a single pulse wave and the AIr for future cardiovascular analysis; (**b**) the pulse wave signal under different preload conditions; (**c**) AIr analysis of different preload conditions; (**d**) photos showing the test environment of the real-time exercising; (**e**) the real-time potassium ion responses and the pulse wave signal with the 40 min exercising and rest.

An important physical signal related to the cardiovascular system is the arterial index of reflection (AIr), which reflects human health status based on analysis of the pulse wave signal during a static test [25,26]. The AIr is calculated as the percentage ratio of the T-wave’s y-value to the P-wave’s y-value. Normally, the AIr is tested under preloading conditions, which can reveal various aspects of an individual’s cardiovascular health. In this study, we attached our DMSE-skin sensor to the fingertip of a recruited subject (male; age: 22 years) to measure his AIr value, and the finger was covered with an airbag to produce different preloads (Figure 5a) by injecting volumes with a syringe. The DMSE-skin sensor was sensitive enough to detect pulse waves precisely under different preloads, as shown in Figure 5b. The AIr values were calculated under varying preloads, with its value decreasing as the preload increased (Figure 5c), indicating potentially healthy arterial stiffness. However, the individual baseline values of the AIr can vary significantly due to various factors, including age, fitness level, and baseline blood pressure. The average AIr for the testing subject was 80%, which is notably higher than the average value of 42% observed in Asian subjects [27,28]. This value suggests that the subject may have been experiencing predictable vascular aging problems, potentially linked to bad habits, such as irregular sleep patterns and a high-carbohydrate diet. Moreover, pulse signal tests were conducted on multiple age groups subjects from 21 years old to 64 years old. The signals obtained demonstrate that as the age increased, the intensity of the P-wave (ΔC_P_) increased and then decreased when the age was over 30 years old. On the other hand, the T-wave exhibited a more linear trend: its intensity of T-wave (ΔC_T_) decreased as the age increased. This indicates that the arterial wall becomes stiffer with age and that the T-wave moves upward and becomes less clear with the increase in age (Supplementary Figure S13) [27,28].

The DMSE-skin sensor’s ability to monitor cardiovascular health was further validated with an exercising test measuring both pulse wave signals and potassium ion responses simultaneously. During the test (Figure 5d), the subject rode an indoor bike for 20 min, followed by a period of rest for another 20 min. The electrochemical workstation and an LCR meter were used to monitor pulse wave signal and K^+^ responses at the same time. The sensor, attached to the fingertip, functioned as the working electrode for both instruments, while Ag/AgCl commercial electrodes attached to the forearm represented the reference and counter electrodes. Figure 5e shows the monitored K^+^ concentration and pulse wave signals during the whole test. During exercise, these two parameters showed different responses in the different stages. During the exercising stages, i.e., “Ramping up” and “Cycling at constant load”, the K^+^-V response showed an increase of around 40 mV because the subject was sweating during these two stages. Then, the subject drank electrolyte water containing K^+^ and continued sweating. After a while, the K^+^-V response curve showed an increase of around 10 mV because of the intake of electrolytes. Eventually, the subject cooled down, and the potential response decreased. Regarding the pulse wave signals, the heart rate increased significantly during exercise and returned to its original level after the rest period (Supplementary Figure S14). These findings show that our sensor can detect pulse wave signals precisely, even when the heart rate undergoes variation. Additionally, the heart rate and capacitance response amplitude in the exercising stages were higher than those in the cool-down stages. All these results demonstrate promising applications for the proposed device in CVD monitoring.

## 4. Discussion

In summary, we developed a dual-model sensing E-skin, relying on a simply stacked electrode structure capable of monitoring mechanical signals, such as pulse wave signals, and electrochemical signal responses simultaneously. The design leverages the skin as a sensing material to achieve high sensitivity in the detection of pressure-capacitance response as well as electrochemical potential–K^+^ concentration. Furthermore, the robustness against interference between these two sensing signals enables the real-time monitoring of the pulse rate and K^+^ in sweat, both of which are vital elements in CVD assessment. The proposed device shows great convenience as in future highly integrated circuits, and our DMSE-skin could help patients monitor their cardiovascular condition without using large devices. We believe that this technology holds great promise for real-world clinical applications. This strategy, which combines multi-sensing models in an epidermal electrode structure, opens up the possibility for the simplification of wearable health monitoring devices and lays the foundation for the future development of multi-model sensors.

## Data Availability

Data are contained within the article.

## Supplementary Materials

The following supporting information can be downloaded at https://www.mdpi.com/article/10.3390/bios15010005/s1. Figure S1: Electrochemical electrode/spacer’s performances in potential in different potassium ion with different valinomycin concentration states; Figure S2: Pulse signals detected on finger using DMSE-skin; Figure S3: Sensitivity performance of different meshes sandpapers’ electrodes; Figure S4: Pressure sensing performances of micro-structured electrode with and without gold coating; Figure S5: Fabrication progress of microstructured electrode and its scanning electron microscope images; Figure S6: Digital photos of DMSE-skin attaching area on skin showing the biocompatibility; Figure S7: Comparison of pressure sensing performance with reported work; Figure S8: Capacitance response under one cycle of 10 kPa pressing-release in 0, 25, and 50 °C; Figure S9: The potential change response to other metabolites like glucose, lactic acid, Na^+,^ and K^+^; Figure S10: Basic noise signals of K^+^-potential response under motion state; Figure S11: Potential changes under gradient K^+^ addition in 0, 25, and 50 °C environment; Figure S12: Comparison of motion caused signals and pulse signals; Figure S13: Using DMSE-skin to record the pulse of subjects (21–64 years old) to analyze their pulse wave signals; Figure S14: Heart rate changes in over 30 min of bicycling.
