# Supplementary material for: A Mechanical–Electrochemical Dual-Model E-Skin for the Monitoring of Cardiovascular Healthcare
Source: Biosensors (Basel). 2024 Dec 26;15(1):5. doi: 10.3390/bios15010005 (PMC11763330; doi:10.3390/bios15010005)
Supplement: Supplementary file 1 [file biosensors-15-00005-s001.zip › biosensors-3330747-supplementary.pdf]

A mechanical-electrochemical dual-model E-skin for the monitoring of cardiovascular healthcare

Jianxiao Fang [1,2,3,4], Yunting Jia [1,4], Zelong Liao [1], Bairui Qi [2] and Tao Huang [1,*]

1. Department of Materials Science and Engineering, Southern University of Science and Technology, Shenzhen, Guangdong, China;
2. Department of Materials Science and Engineering, Central South University, Changsha, Hunan, China;
3. Department of Materials, The University of Manchester, Manchester, Greater Manchester, England;
4. These authors contribute equally.

*. Correspondence and requests for materials should be addressed to Tao Huang (email: huangt@sustech.edu.cn)

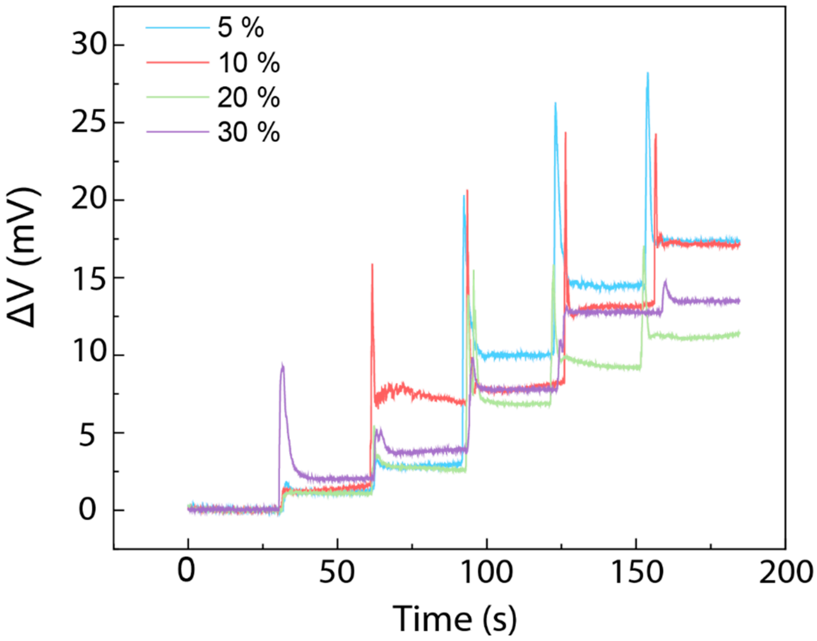

Supplementary Figure S1 | Electrochemical potential response to K^+ using 5%, 10%, 20% and 30% weight content of valinomycin in spacer.

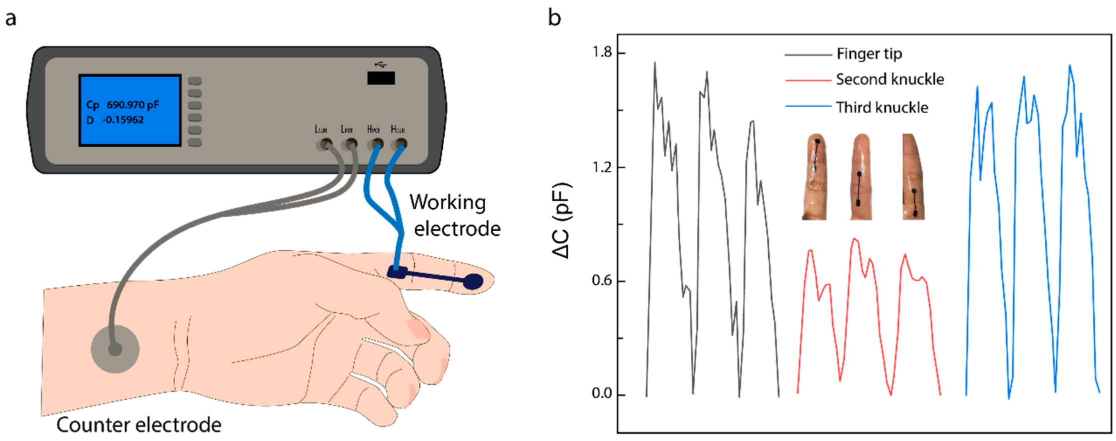

Supplementary Figure S2 | Pulse signals detected on finger using DMSE-skin. a. Testing setup for capacitance recording; **b.** Pulse wave signals recorded on different knuckles of the finger.

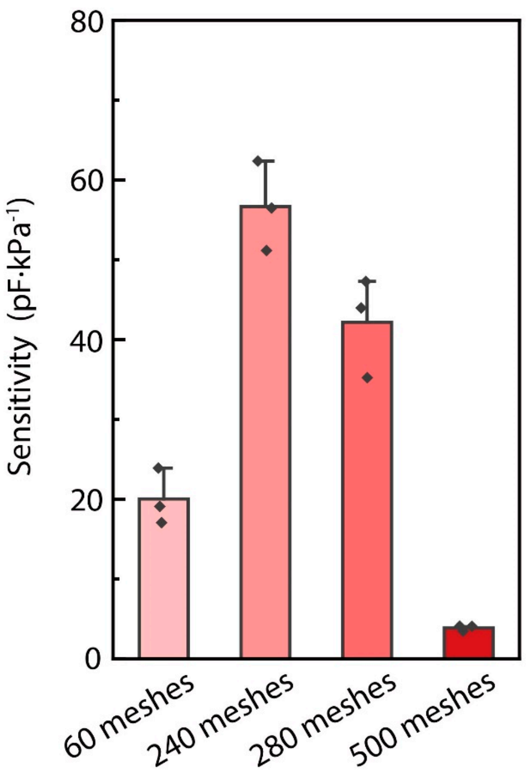

Supplementary Figure S3 | Sensitivity performance of electrode using different microstructure templated from 60, 240, 280, and 500 meshes sandpapers. The results illustrated that the 240 meshes sandpaper exhibits the best sensitivity performance among other sizes.

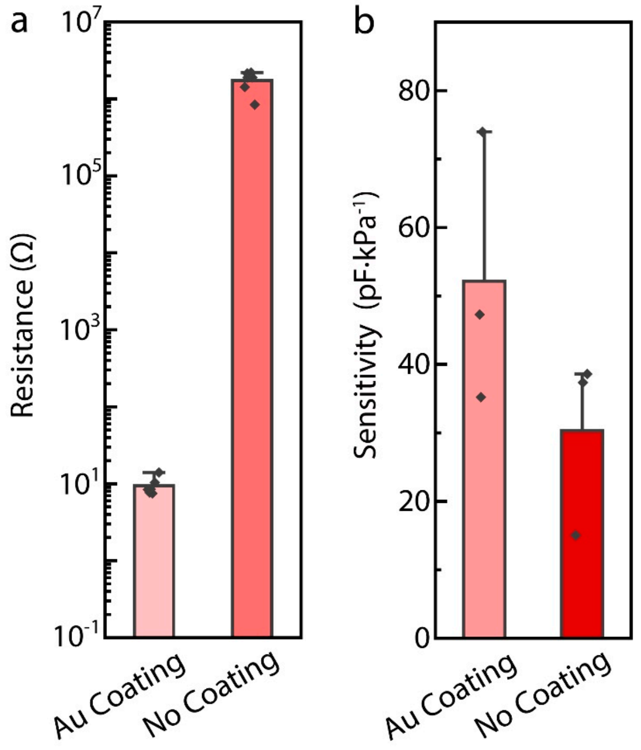

Supplementary Figure S4 | Pressure sensing performances of micro-structured electrode with and without gold coating. **a.** Resistance with and without Au coating; **b.** Sensitivity with and without Au coating.

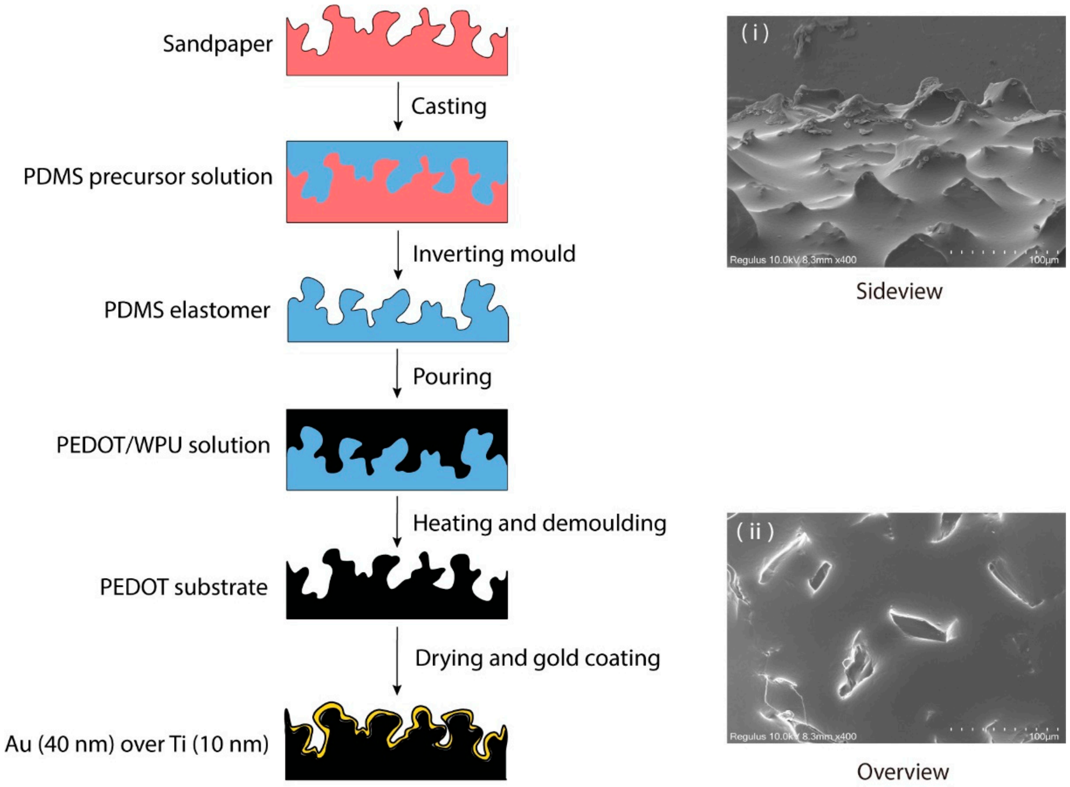

Supplementary Figure S5 | Fabrication progress of microstructured electrode and its scanning electron microscope images.

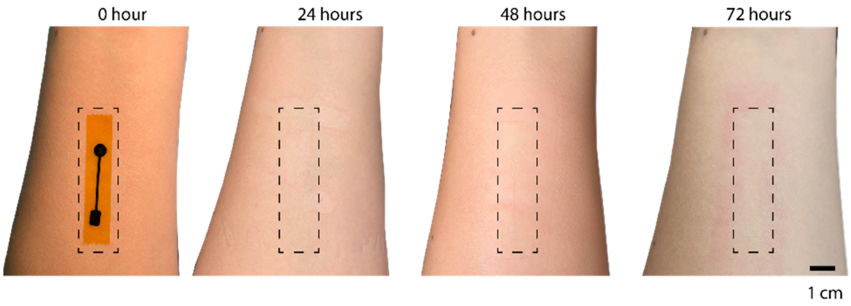

Supplementary Figure S6 | Digital photos of DMSE-skin attaching area on skin showing the biocompatibility.

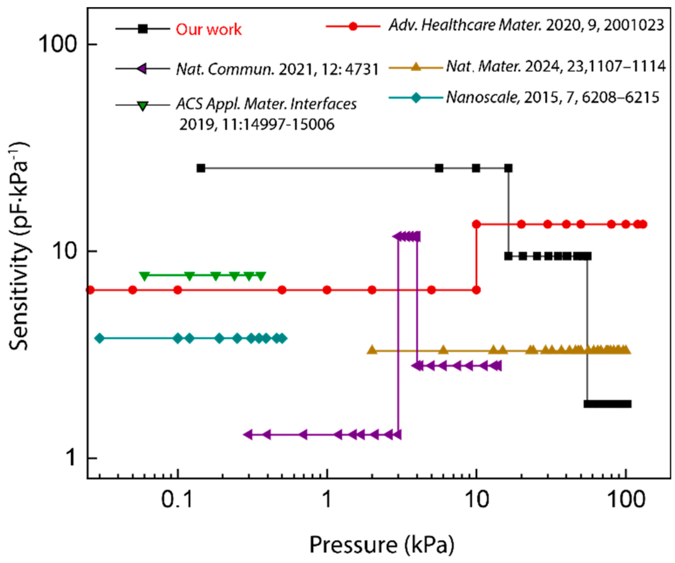

Supplementary Figure S7 | Comparison of pressure sensing performance with reported work.

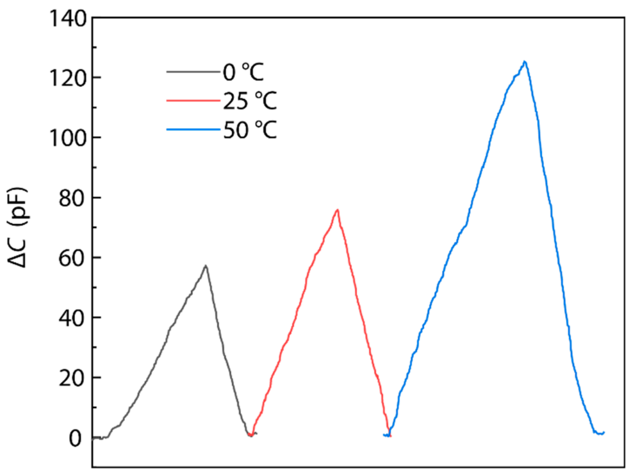

Supplementary Figure S8 | Capacitance response under one cycle of 10 kPa pressing-release in 0, 25, and 50 °C

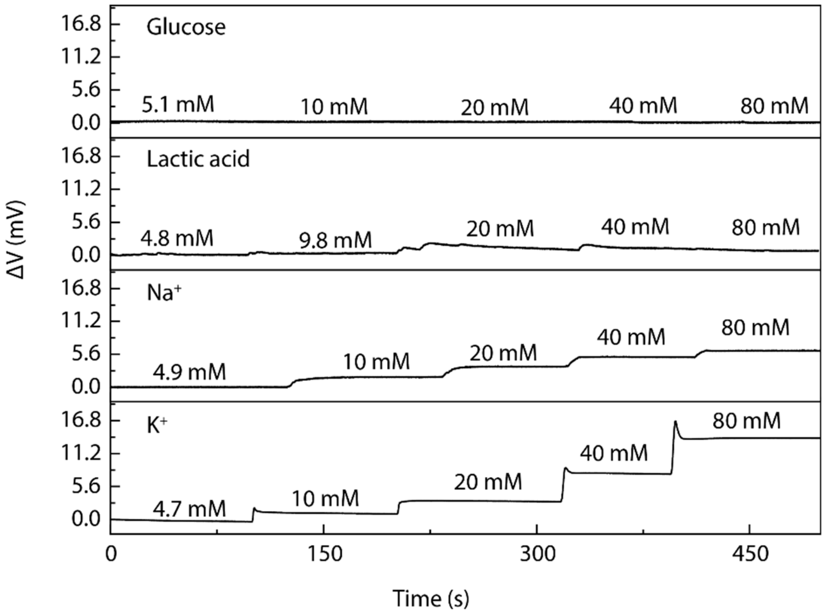

Supplementary Figure S9 | The potential change response to other metabolites like glucose, lactic acid, Na^+ and K^+.

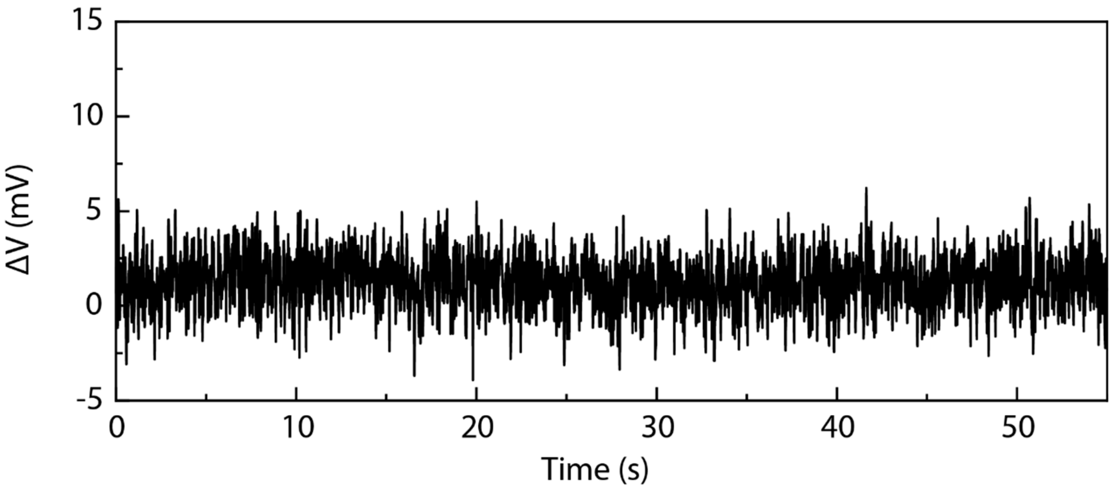

Supplementary Figure S10 | Basic noise signals of K^+-potential response under motion state.

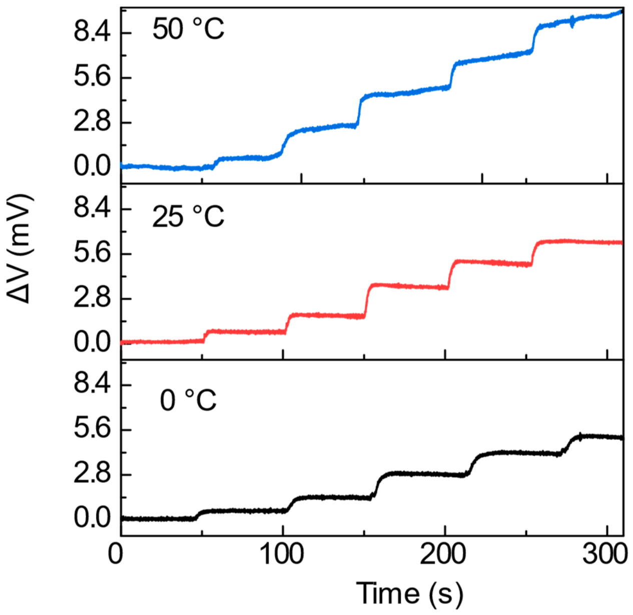

Supplementary Figure S11 | Potential changes under gradient K^+ addition in 0, 25, and 50 °C environment

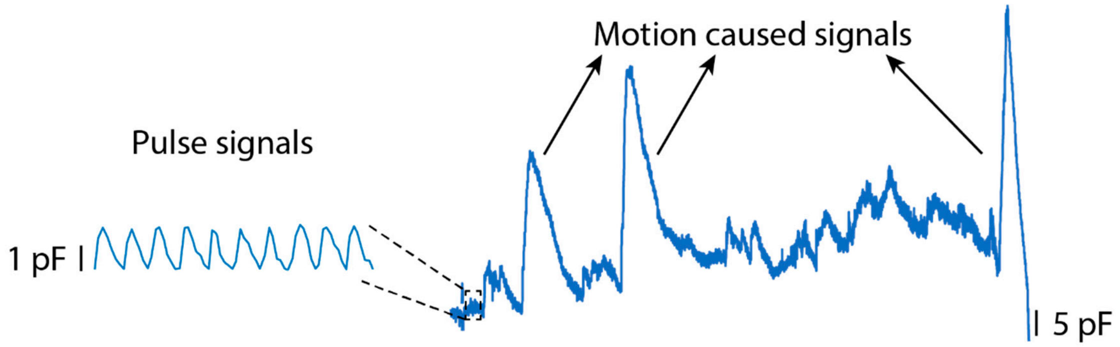

Supplementary Figure S12 | Comparison of motion caused signals and pulse signals.

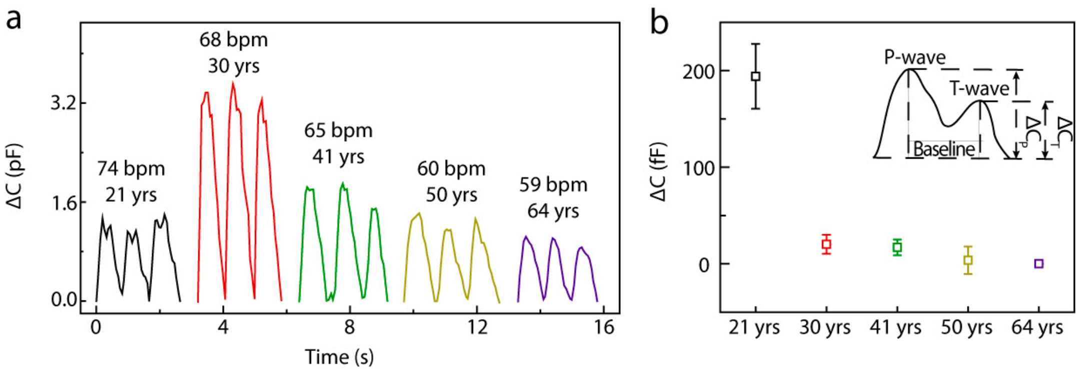

Supplementary Figure S13 | Using DMSE-skin to record the pulse of subjects (21-64 years old) to analyze their pulse wave signals. **a.** Capacitance signals of 5 subjects' pulse wave with their ages and heart rates; **b.** Comparison of 5 subjects' T-wave intensity.

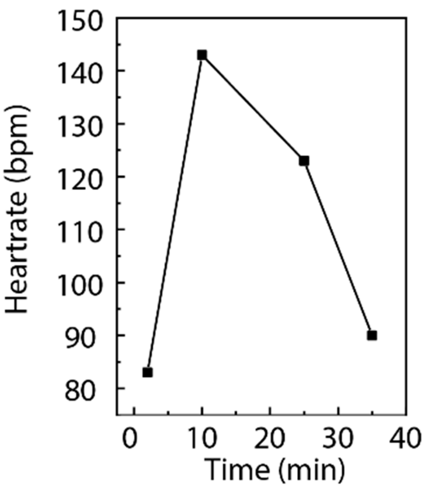

Supplementary Figure S14 | Heartrate changes in over 30 min of bicycling.
